# Transcriptome analysis of the brown rot fungus *Gloeophyllum trabeum* during lignocellulose degradation

**DOI:** 10.1371/journal.pone.0243984

**Published:** 2020-12-14

**Authors:** Kiwamu Umezawa, Mai Niikura, Yuka Kojima, Barry Goodell, Makoto Yoshida

**Affiliations:** 1 Department of Environmental and Natural Resource Science, Tokyo University of Agriculture and Technology, Tokyo, Japan; 2 Department of Applied Biological Chemistry, Kindai University, Nara, Japan; 3 Department of Microbiology, University of Massachusetts Amherst, Amherst, Massachusetts, United States of America; Austrian Institute of Technology, AUSTRIA

## Abstract

Brown rot fungi have great potential in biorefinery wood conversion systems because they are the primary wood decomposers in coniferous forests and have an efficient lignocellulose degrading system. Their initial wood degradation mechanism is thought to consist of an oxidative radical-based system that acts sequentially with an enzymatic saccharification system, but the complete molecular mechanism of this system has not yet been elucidated. Some studies have shown that wood degradation mechanisms of brown rot fungi have diversity in their substrate selectivity. *Gloeophyllum trabeum*, one of the most studied brown rot species, has broad substrate selectivity and even can degrade some grasses. However, the basis for this broad substrate specificity is poorly understood. In this study, we performed RNA-seq analyses on *G*. *trabeum* grown on media containing glucose, cellulose, or Japanese cedar (*Cryptomeria japonica*) as the sole carbon source. Comparison to the gene expression on glucose, 1,129 genes were upregulated on cellulose and 1,516 genes were upregulated on cedar. Carbohydrate Active enZyme (CAZyme) genes upregulated on cellulose and cedar media by *G*. *trabeum* included glycoside hyrolase family 12 (GH12), GH131, carbohydrate esterase family 1 (CE1), auxiliary activities family 3 subfamily 1 (AA3_1), AA3_2, AA3_4 and AA9, which is a newly reported expression pattern for brown rot fungi. The upregulation of both terpene synthase and cytochrome P450 genes on cedar media suggests the potential importance of these gene products in the production of secondary metabolites associated with the chelator-mediated Fenton reaction. These results provide new insights into the inherent wood degradation mechanism of *G*. *trabeum* and the diversity of brown rot mechanisms.

## Introduction

Brown rot Basidiomycota are the dominant wood decay fungi in northern coniferous forests and they are also the primary cause of decay failure in wooden structures. Because of their ability to deconstruct woody biomass in a unique manner, they have attracted attention for use by biorefineries for the generation of bio-based fuels and chemicals.

Brown rot fungi degrade plant cell wall polysaccharides such as cellulose and hemicelluloses, but do not metabolize lignin, although they extensively depolymerize and modify it [1]. It has been reported by several groups that brown rot fungi use a two-step process where an incipient oxidative radical system opens the lignocellulose structure followed by an enzymatic saccharification system using Carbohydrate-Active enZymes (CAZymes) [2–4]. The brown rot fungi lack class II peroxidases, which are essential enzymes for lignin metabolism, and fewer glycoside hydrolases (GHs) are involved in cellulose degradation compared to white rot fungi [5,6]. Most brown rot fungi also lack cellobiohydrolases and cellobiose dehydrogenases, which are known to play critical roles in crystalline cellulose degradation in other filamentous fungi [7]. They, however, possess endoglucanases, β-glucosidases, and lytic polysaccharide monooxygenases, which work as cellulose degrading enzymes [6,8].

The incipient oxidative system, known as the chelator-mediated Fenton (CMF) reaction [9,10], triggers the formation of hydroxyl radicals (·OH) which attack the wood cell wall associated at sites where iron-binding occurs. Prior research suggests that the structure of wood cell walls is opened by CMF action to allow subsequent enhanced action by enzymes [11]. In the CMF reaction, iron will be reduced inside cell walls by low molecular weight (LMW) hydroquinones and related metabolites produced by the fungus. Quinone reductases, Fe^3+^-reducing glycopeptides, and hemoproteins belonging to the Auxiliary Activities family 8 (AA8) have been proposed to produce Fe^2+^ by reduction of Fe^3+^ [12–14], but these hypotheses for the generation of reduced iron is still under discussion because the enzyme (or large proteins or peptide complexes) would need to be active deep within plant cell walls. Because these enzymes and larger peptide complexes are too large to penetrate the dense nanostructure of the intact lignified plant cell wall, enzymatic action deep within wood cell walls is not possible. Iron reduction by low molecular weight hydroquinones and similar phenolic iron reductants secreted by the fungus as described in the CMF mechanism [2], or even by lignin fragments on nanosurfaces within the cell wall [15], is a more likely source for reduced iron required within the plant cell wall. In the CMF reaction, H_2_O_2_ is proposed to be produced by both by LMW compounds within the wood cell wall, and extracellular enzymes [2]. Alcohol oxidases and copper radical oxidases are some of the enzymes proposed to be implicated in this H_2_O_2_ production [16,17], but considering the reactivity of H_2_O_2_, a source of H_2_O_2_ that was produced within plant cell walls, as opposed to an enzymatic source produced near the hyphae, would be more efficient. The prominent pathway for the generation of H_2_O_2_ in brown rot fungi has been described as iron-dependent hydroquinone autoxidation during decay [18]. Such non-enzymatic pathways for the generation of H_2_O_2_ in other systems containing phenolic compounds are well known [19,20]. After action by the CMF mechanism to open the structure of the wood cell wall, enzymatic saccharification system then occurs to depolymerize polysaccharides, ultimately to monosaccharides.

Brown rot fungi have generally been considered as gymnosperm specialists. Recently, however, Krah et al. [21] suggested that different brown rot fungi may show different substrate selectivity among the phyla. The brown rot Polyporales, for example, have been considered to be generalists regarding their ability to attack both gymnosperms and angiosperms, whereas the Gloeophyllales and Boletales have been considered as primarily gymnosperm specialists. Some Gloeophyllales species, however, such as *Gloeophyllum trabeum*, are able to degrade some grass substrates while some Antrodia clade fungi degrade grasses inefficiently [22,23]. Presley et al. [24] also demonstrated different hemicellulase activities between two representative brown rot fungi, with higher xylanase activity in *G*. *trabeum* and higher mannanase activity in *Serpula lacrymans*. These facts suggest that brown rot fungi from different orders and clades may have different wood degradation mechanisms and, as such, distinctions in sub-types of brown rot degradation may become more apparent as we learn more about the mechanisms these fungi employ. Transcriptomic analysis can help clarify the basis for the different plant assimilation properties. Therefore, in this study, we performed RNA-seq analysis of *G*. *trabeum* grown on media containing glucose, cellulose, or conifer wood (*Cryptomeria japonica*; Japanese cedar) as the sole carbon source to clarify the details of gene expression during lignocellulose degradation. This research represents the first transcriptomic analysis of *G*. *trabeum*, comparing gene expression of this fungus when grown on different substrates.

## Materials and methods

### Culture conditions

*Gloeophyllum trabeum* strain NBRC 6430 was maintained on potato dextrose agar (Nihon Pharmaceutical Co., Japan) at 26°C. Agar plugs covered with mycelium were transferred to 500 ml Erlenmeyer flasks containing 200 ml Highley’s medium (2 g NH_4_NO_3_, 2 g KH_2_PO_4_, 0.5 g MgSO_4_·7H_2_O, 0.036 mg CuSO_4_·5H_2_O, ZnSO_4_·7H_2_O, FeSO_4_·7H_2_O, 0.1 g CaCl_2_·2H_2_O, 0.31 mg MnCl_2_·4H_2_O, 0.018 mg (NH_4_)_6_Mo_7_O_24_·4H_2_O, 0.57 mg H_3_BO_4_, 1 g thiamin hydrochloride per liter H_2_O) supplemented with 0.5% glucose, microcrystalline cellulose (Avicel; Merck, Germany), or *Cryptomeria japonica* (Japanese cedar) as the sole carbon source [25]. It is recognized that the addition of iron and other transition metals in media will repress the expression of LMW iron-binding metabolites; however, in this work, our focus was on enzyme expression. Cellulose and cedar wood flour were nanofibrillated by aqueous counter collision treatment using an ejection pressure of 200 MPa with 60 cycle repetition times. *G*. *trabeum* typically grows better in stationary culture or in bioreactors with high surface area to allow the mycelial immobilization, in our research the fungus was cultivated in flasks on a rotary shaker (160 rpm) at 27°C for 3 days (glucose) or 5 days (cellulose and cedar) causing them to form spherical mycelial “balls” during growth. The cultivation periods were determined based on the growth rate of mycelium in each carbon source. After cultivation, mycelia were collected by filtration, then immediately frozen and stored at -80°C for subsequent RNA-seq analysis.

### RNA extraction and sequencing

Total RNA was isolated from frozen samples using a RNeasy Plant Mini Kit (Qiagen, Germany). RNA concentration and quality were determined using TapeStation (Agilent Technologies, CA, USA). The RNA integrity numbers (RIN) for each sample were determined and RIN > 9 were used for RNA sequencing. Strand-specific cDNA libraries for RNA-seq were constructed using a TruSeq Stranded mRNA Library Prep Kit (Illumina, CA, USA) using *G*. *trabeum* samples grown on the three carbon sources (above) with three biological replicates. These libraries were sequenced by HiSeq2000 (Illumina, CA, USA) with 2 × 100 bp paired-end readings. The construction of cDNA libraries and the subsequent RNA-seq was carried out using a custom service (Eurofins Genomics, Japan).

### RNA-seq analysis

The raw reads were trimmed and filtered to remove low-quality bases using FaQCs v 1.34 with a minimum phred quality score of 20 and a minimum length of 50 bp [26]. Clean reads were used to pseudoalign to the filtered models' sequences obtained by JGI (https://genome.jgi.doe.gov/ Glotr1_1), using Kallisto v. 0.44.0 (option—bias—rf-stranded -b 100) [27]. Kallisto output was used as input for Sleuth v. 0.29.0 to estimate normalized transcripts per million (TPM) and perform differentially expressed gene (DEG) analyses [27]. Genes with q-value < 0.01 by likelihood-ratio tests (LRTs) and fold change ≥ 2 were taken as DEGs. The cluster dendrogram was generated in R using plot(hclust(dist(x))) with the average linkage method for clustering and Spearman's correlation as the distance metric. CAZymes were predicted using dbCAN, the automated CAZyme prediction program [28]. For the carbohydrate esterase family 1 (CE1) gene, other esterase genes were annotated as CE1 genes. Therefore, these annotations were removed manually. In addition, CE10 family annotations were removed because the CAZy database has withdrawn this families’ annotations. All raw fastq files were deposited in the GEO (Gene Expression Omnibus) database (accession number GSE 155681).

## Results

### Sequencing statistics and cluster dendrogram for RNA-seq samples

**Fig 1:**
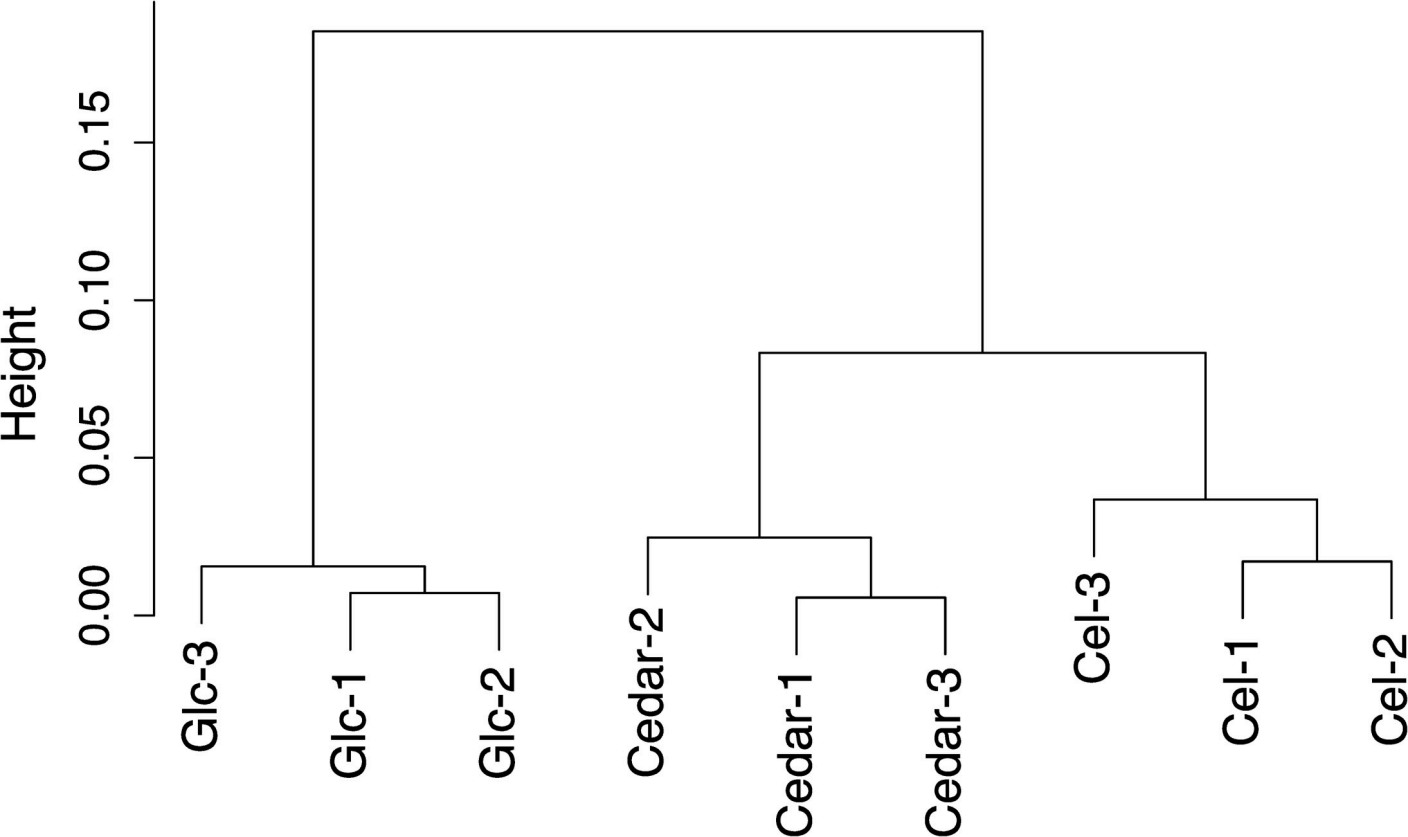
Cluster dendrogram of the entire gene expression data set. Spearman's correlation values were converted into distance coefficients to define the height of the dendrogram.

A total of 9 RNA libraries were sequenced with the number of reads ranging from 14.3–24.8 million paired-end reads (S1 Table). 95.9–97.7% of the reads passed the quality control and 61.3–78.1% of the clean reads were pseudoaligned to the reference transcripts (S1 Table). Hierarchical clustering analysis was carried out to assess the relatedness of *G*. *trabeum* fungal growth on the three different carbon source samples, glucose, cellulose, and Japanese cedar (Fig 1). The replicates for all three of the conditions were highly reproducible. The expression profiles of cellulose- and cedar-cultured fungi were closely correlated, while the glucose-cultured fungi clustered distantly. This suggested that both cellulose and cedar wood triggered the induction of a unique plant cell wall degrading system in *G*. *trabeum*.

### Identification of differentially expressed genes

**Fig 2:**
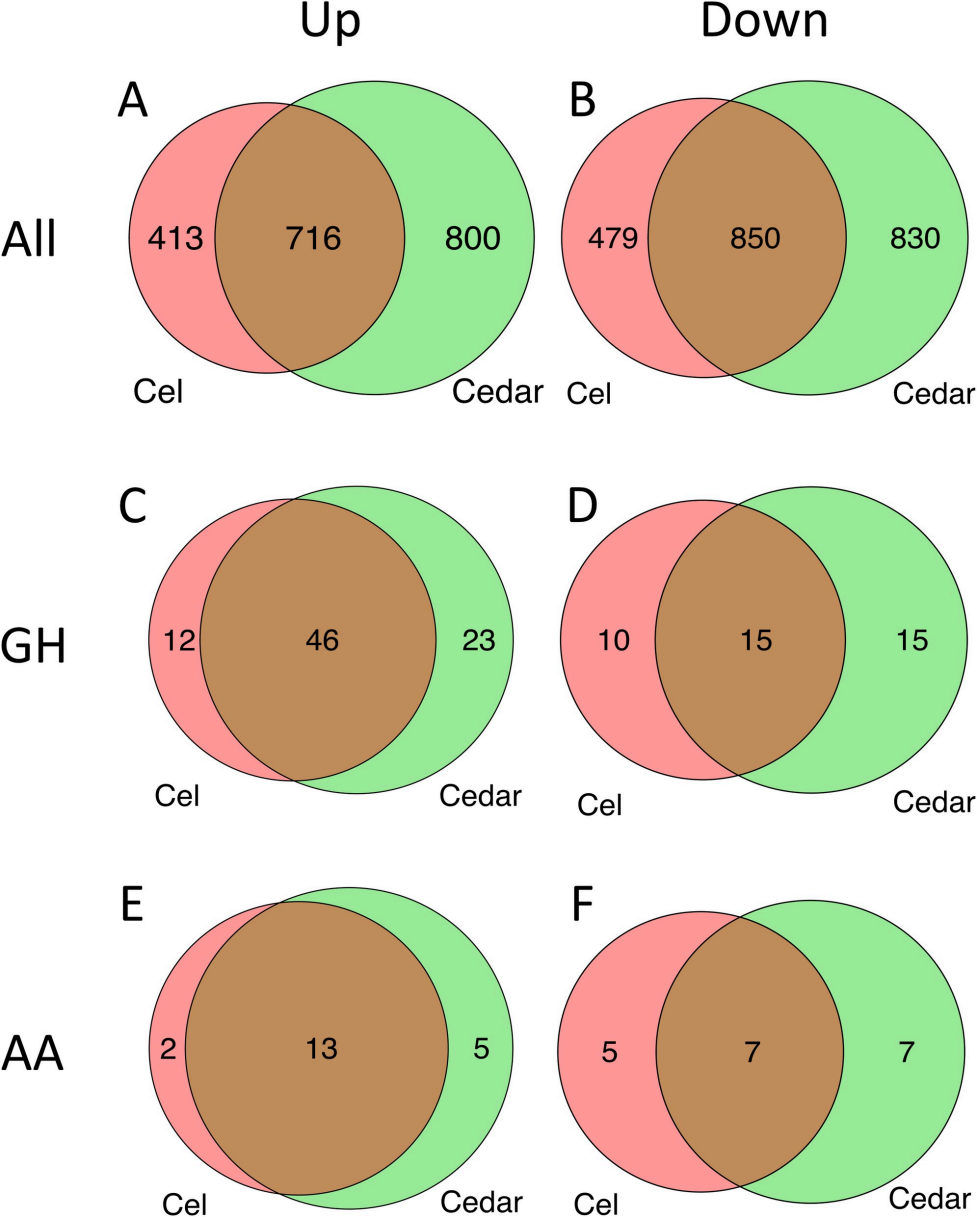
Genes expressed differentially when *G*. *trabeum* was grown on cellulose (red) and cedar (green) compared to when the fungus was grown on glucose. Number of upregulated (A, C, E) and downregulated (B, D, F) genes from all gene models (A, B), GH (C, D), and AA (E, F).

We compared the transcriptomic data between glucose, cellulose, and cedar samples to assess differential gene expression. Of the 11846 JGI *G*. *trabeum* gene models, 1,129 and 1,516 genes, respectively, were determined to be upregulated (q < 0.01, fold change ≥ 2) when grown on cellulose and cedar relative to glucose (Fig 2A). The upregulated genes overlap considerably, and 716 genes were shared in these samples. Conversely, 1,329 and 1,680 genes, respectively, were downregulated when the fungus was grown on cellulose and cedar relative to glucose (Fig 2B). Of these downregulated genes, 850 were consistently downregulated when the fungus was grown on either cellulose or cedar media.

CAZymes are crucial enzymes for the degradation of extracellular carbohydrate sources. The result of the CAZyme annotation tool dbCAN showed that *G*. *trabeum* encodes 366 CAZymes, which included 207 GHs, 71 glycosyltransferases (GTs), 10 polysaccharide lyases (PLs), 22 CEs, and 57 AAs. Of the GH genes, 58 and 69 genes were upregulated on cellulose and cedar cultures respectively, and 49 genes of them were shared in both conditions (Fig 2C). Conversely, 25 and 30 GH genes were downregulated when grown on cellulose and cedar, respectively, and 15 genes of these genes were shared under both conditions (Fig 2D).

With respect to the AA genes, 15 and 18 genes were upregulated on cellulose and cedar respectively, with 13 of these genes shared in both conditions (Fig 2E). Nearly as many AA genes, 12 and 14 respectively, were downregulated when *G*. *trabeum* was grown on cellulose and cedar. Seven of these downregulated genes were shared when the fungus was grown either on cellulose or cedar (Fig 2F). Specific information on the differentially expressed CAZyme genes is provided in S2 Table.

#### Cellulose and hemicellulose degradation

The GH5_5 and GH12 endoglucanase families are known to be active in the degradation of cellulose by brown rot fungi [29]. Two GH5_5 endo-β-1,4-glucanase genes from *G*. *trabeum* (Glotr_57704, Glotr_63180) were significantly upregulated on both cellulose and cedar relative to glucose (Table 1). Glotr_63180 was observed to be the third most highly upregulated gene on cellulose (ca. 2,401-fold). GH12 enzymes act on β-1,4-glucans in cellulose and xyloglucans. The GH12 gene Glotr_138821 was significantly upregulated on both cellulose and cedar, but was especially notable on cellulose (ca. 773-fold) (Table 1). AA9 lytic polysaccharide monooxygenases (LPMO9s) are known to oxidatively cleave both cellulose and hemicellulose chains. In this study, *GtLPMO9B* (Glotr_63531) was significantly upregulated on cellulose and cedar, while *GtLPMO9A* (Glotr_45893, previously reported as a xyloglucan degrading enzyme [30]) was downregulated on both these media (Table 1 and S3 Table). GH131 has been assigned to be a β-glucanase which has activity against β-1,3/1,6- and β-1,4-linked glucan and is thought to be involved in cellulose degradation [31]. The only GH131 gene, Glotr_106470, was significantly upregulated on both cellulose and cedar cultures (Table 1). GH3 enzymes act on the non-reducing end of various β-1,4-glycans of cellulose and hemicellulose. *G*. *trabeum* has ten GH3 genes and five of these were significantly upregulated on both cellulose and cedar (Table 1). Two of the remaining GH3 genes were uniquely upregulated on cellulose whereas only one was upregulated on cedar (Table 1 and S3 Table). GH1 genes produce intracellular enzymes that target the terminal β-O-1,4 glycosidic residues in both cellulose and hemicellulose. In *G*. *trabeum*, one of the five GH1 genes (Glotr_141319) was significantly upregulated on both cellulose and cedar (Table 1 and S3 Table).

**Table 1:** *G*. *trabeum* genes encoding putative cellulose and hemicellulose degrading enzyme genes.

		TPM(Average)^a^			Cel/Glc^b^		Cedar/Glc^b^			
ID	Putative function	Glc	Cel	Cedar	Ratio	Q value	Ratio	Q value	Up^c^	Down^c^
57704	GH5_5 endo-β-1,4-glucanase	15.7	646.9	96.9	41.2	0.001	6.2	0.008	C, S	
63180	GH5_5 endo-β-1,4-glucanase	3.4	8279.2	246.0	2401.3	0.000	71.4	0.002	C, S	
62344	GH12 endo-β-1,4-glucanase	0.2	0.4	1.4	2.2	0.200	8.7	0.006	S	
138821	GH12 endo-β-1,4-glucanase	4.8	3698.0	62.3	772.8	0.000	13.0	0.001	C, S	
45893	AA9 lytic polysaccharide monooxygenase	20.5	1.2	1.6	0.1	0.001	0.1	0.000		C, S
63531	AA9 lytic polysaccharide monooxygenase	27.2	8353.6	798.7	306.6	0.000	29.3	0.005	C, S	
106470	GH131 β-glucanase	6.9	433.7	125.1	62.9	0.003	18.1	0.003	C, S	
44548	GH3 exo-β-glycosidase	2.1	15.5	5.4	7.5	0.041	2.6	0.000	S	
54923	GH3 exo-β-glycosidase	5.0	63.3	13.2	12.7	0.000	2.6	0.001	C, S	
69843	GH3 exo-β-glycosidase	3.5	6.9	8.7	2.0	0.002	2.5	0.000	S	
71534	GH3 exo-β-glycosidase	8.6	70.7	23.1	8.3	0.000	2.7	0.000	C, S	
72986	GH3 exo-β-glycosidase	11.8	727.0	214.5	61.8	0.000	18.2	0.000	C, S	
122002	GH3 exo-β-glycosidase	4.2	11.6	2.3	2.8	0.001	0.5	0.002	C	
141438	GH3 exo-β-glycosidase	10.8	469.8	153.6	43.6	0.000	14.2	0.000	C, S	
141319	GH1 β-glucosidase	4.8	231.8	24.2	48.4	0.000	5.0	0.001	C, S	
46499	GH10 β-1,4-xylanase	6.9	2320.5	106.5	337.1	0.000	15.5	0.003	C, S	
140289	GH10 β-1,4-xylanase	2.2	1026.3	138.0	456.5	0.000	61.4	0.002	C, S	
110405	GH5_7 β-mannanase	8.8	154.2	24.3	17.4	0.006	2.8	0.028	C	
135369	GH5_7 β-mannanase	7.7	198.5	56.5	25.9	0.000	7.4	0.000	C, S	
117128	CE1 esterase	4.8	317.2	70.3	66.3	0.000	14.7	0.000	C, S	
6650	GH28 endopolygalacturonase	1.0	0.8	2.5	0.8	0.065	2.6	0.001	S	
54367	GH28 endopolygalacturonase	1.3	3.9	2.8	3.0	0.001	2.1	0.002	C, S	
138836	GH28 endopolygalacturonase	0.8	0.6	1.9	0.8	0.162	2.4	0.002	S	
141341	GH28 endopolygalacturonase	3.9	6.6	8.0	1.7	0.013	2.1	0.000	S	
77537	CE8 pectin metylesterase	2.0	5.5	9.6	2.7	0.013	4.8	0.000	S	

^a^Mean TPM value for each condition (n = 3).

^b^Ratio of TPM value and Q value by LRTs between cellulose and glucose, and cedar and glucose.

^c^Genes determined as upregulated (Up) or downregulated (Down). C: Cellulose, S: Cedar.

As for hemicellulose-specific degrading enzymes, two of the three GH10 β-1,4-xylanase genes (Glotr_46499, Glotr_140289) were upregulated on cellulose and cedar (Table 1 and S3 Table). *G*. *trabeum* has two GH5_7 β-mannanase genes, with Glotr_135369 upregulated on both cellulose and cedar, while Glotr_110405 was upregulated only on cellulose (Table 1). CE1 esterases have important roles in arabinoxylan degradation [32]. In this study, Glotr_117128—the only CE1 in *G*. *trabeum*, was upregulated on both cellulose and cedar (Table 1).

Relative to pectin degradation by *G*. *trabeum*, upregulation of four of the ten GH28 endopolygalacturonase genes and one CE8 pectin metylesterase gene occurred on cedar, although the expression levels of these genes were low (Table 1 and S3 Table).

#### Biosynthesis of LMW iron-binding metabolites

In incipient brown rot decay, the oxidative CMF reaction system initiates deconstruction of the wood cell wall by reducing Fe^3+^ within the wood cell wall. Simultaneously, the generation of H_2_O_2_ or similar oxidants also must occur, either within the cell wall, or in a manner such that these oxidants can diffuse deep within the cell wall to the location of iron and the active site of lignocellulose depolymerization. In regard to iron reduction, LMW compounds and some enzymes have been proposed to carry out this reaction [2,33]; however, enzymes are unable to penetrate deeply within the cell wall until late stages of decay. Hydroquinones and related iron-binding metabolites (referred to as LMW chelators in this paper) from the fungi do have the capability to penetrate the intact wood cell wall structure and have been shown to reduce iron during brown rot fungal degradation of wood cell walls [34,35]. Although little is known about the genes involved in the fungal chelator production, genes related to secondary metabolism have been proposed to be involved in the production of these LMW chelators.

Polyketide synthases (PKSs) produce catechol and quinone compounds which, if expressed and secreted extracellularly during decay, could be the source of the LMW chelators/hydroquinones in the CMF reaction [6]. In this study, *G*. *trabeum* showed no upregulation of PKS genes on cellulose and cedar media (S4 Table).

Terpene synthases (TSs) produce terpenoid compounds and these compounds might also potentially be involved in fungal siderophore (iron chelator) production [36]. *G*. *trabeum* significantly upregulated two TS genes when grown on cellulose or cedar media, while two other TS genes were upregulated only on cellulose media (Table 2 and S4 Table).

**Table 2:** *G*. *trabeum* LMW iron-binding metabolites synthesis associated genes.

		TPM(Average)^a^			Cel/Glc^b^		Cedar/Glc^b^			
ID	Putative function	Glc	Cel	Cedar	Ratio	Q value	Ratio	Q value	Up^c^	Down^c^
116237	Terpene synthase	6.9	129.5	38.5	18.8	0.001	5.6	0.000	C, S	
131990	Terpene synthase	2.7	13.3	7.4	4.9	0.005	2.7	0.000	C, S	
79917	Terpene synthase	2.4	8.2	4.7	3.5	0.002	2.0	0.016	C	
103889	Terpene synthase	150.1	1701.2	805.6	11.3	0.005	5.4	0.014	C	
66940	AA6 1,4-benzoquinone reductase	19.8	19.2	9.8	1.0	1.000	0.5	0.000		S
82342	AA6 1,4-benzoquinone reductase	323.9	91.1	117.5	0.3	0.003	0.4	0.002		C, S
101783	AA6 1,4-benzoquinone reductase	14.0	5.0	8.1	0.4	0.012	0.6	0.015		
130426	AA1_1 laccase	0.8	17.5	11.0	21.0	0.000	13.2	0.000	C, S	
58158	Fe^3+^-reducing glycopeptide (GLP1)	175.0	566.1	210.4	3.2	0.000	1.2	0.001	C	
101715	Fe^3+^-reducing glycopeptide (GLP1)	3723.8	29312.9	13218.4	7.9	0.002	3.5	0.000	C, S	
104526	Fe^3+^-reducing glycopeptide (GLP1)	1.1	197.4	277.5	181.3	0.001	254.8	0.000	C, S	
132851	Fe^3+^-reducing glycopeptide (GLP1)	74.3	5045.9	408.7	67.9	0.001	5.5	0.001	C, S	

^a^Mean TPM value for each condition (n = 3).

^b^Ratio of TPM value and Q value by LRTs between cellulose and glucose, and cedar and glucose.

^c^Genes determined as upregulated (Up) or downregulated (Down). C: Cellulose, S: Cedar.

**Fig 3:**
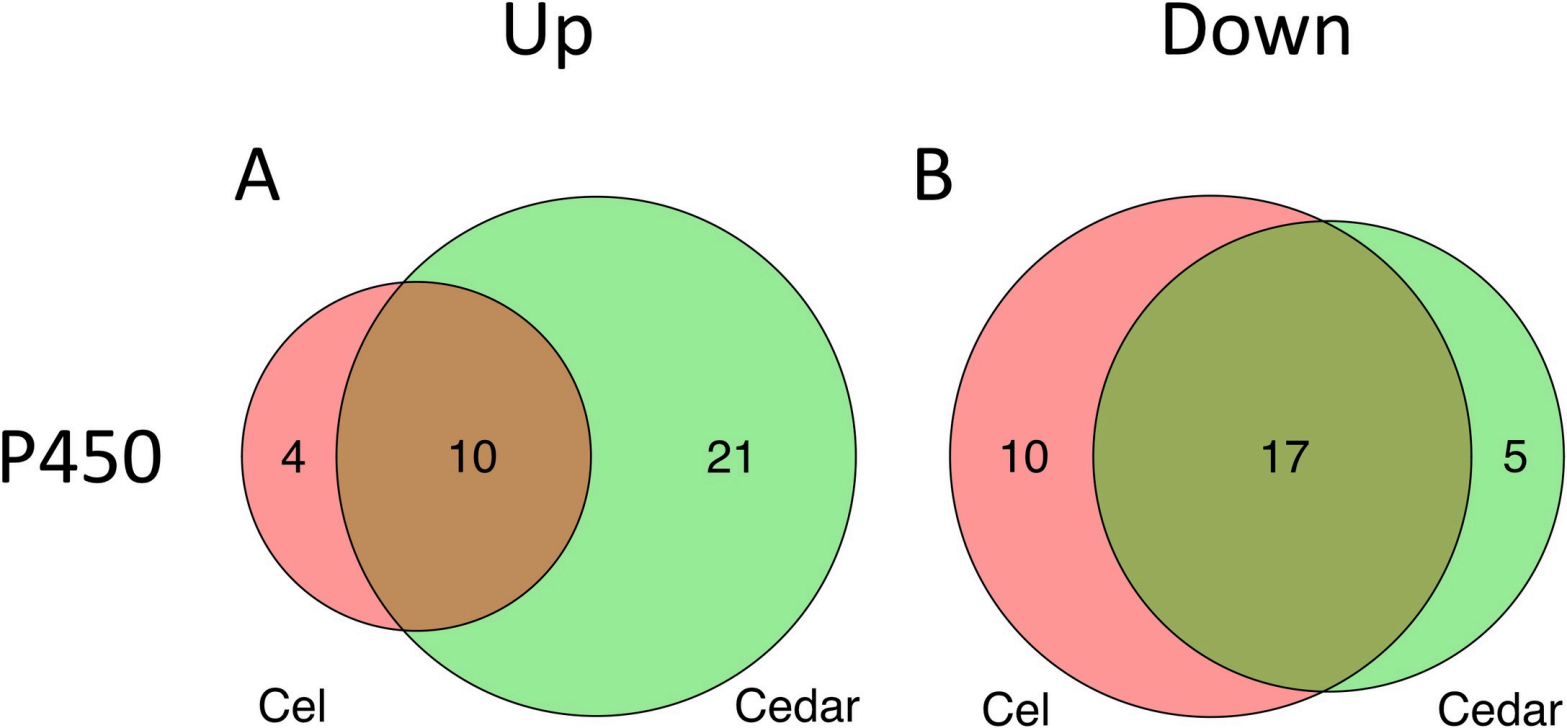
Differentially expressed cytochrome P450 genes when *G*. *trabeum* was grown on cellulose (red) and cedar (green) compared to when the fungus was grown on glucose. Number of upregulated (A) and downregulated (B) genes.

The cytochrome P450 superfamily of monooxygenases can play various roles in secondary metabolism, and in wood decay fungi these roles include the degradation of lignin and xenobiotic compounds [37,38]. Additionally, P450 has been suggested to be involved in pathways for the production of siderophores in bacteria and the production of phenolic compounds in brown rot fungi [38–40]. We postulate that siderophores and/or related compounds produced by P450 enzymes might function as chelators in the CMF reaction in brown rot fungi. In the *G*. *trabeum* genome, 114 P450 genes were annotated. In this study, ten of these genes were upregulated on both cellulose and cedar (Fig 3 and S6 Table). An additional 21 genes were uniquely upregulated on cedar, while an additional four genes were upregulated only on cellulose (Fig 3 and S6 Table).

#### Enzymes potentially involved in LMW catechol redox reactions

AA6 1,4-benzoquinone reductases are able to reduce 2,5-dimethoxy-1,4-benzoquinone (2,5-DMBQ) and have been proposed to be involved in iron reduction in the CMF reaction [12]. In this study a AA6 gene, *QRD1*(Glotr_82342) was downregulated on both cellulose and cedar relative to glucose (Table 2). *QRD2* (Glotr_101783) and another AA6 gene (Glotr_66940) also were not upregulated on cellulose and cedar cultures (Table 2). AA1_1 laccases, which oxidize methoxyhydroquinones to semiquinones (better reductants of Fe^3+^), have also been proposed to function in CMF mechanisms [41]. In this study, one of the four AA1_1 gene (Glotr_130426) was upregulated on both cellulose and cedar media (Table 2 and S5 Table). An Fe^3+^-reducing glycopeptide (GLP) has also been proposed as the Fe^3+^ reductant in the CMF reaction in brown and white rot fungi [13] and three genes potentially related to GLP production (Glotr_101715, Glotr_130687, Glotr_104526) were found to be upregulated on cellulose and cedar media, with an additional gene (Glotr_58158) upregulated only on cellulose media (Table 2).

#### Oxalate and H_2_O_2_ production

Oxalic acid has been suggested to play a role in chelating and solubilizing iron from Fe (oxyhydr)oxide complexes in wood while releasing iron under appropriate microsite conditions to promote the CMF reaction. Two enzymes are generally considered to be involved in oxalate biosynthesis; oxaloacetate acetylhydrolase (OAH) and glyoxylate dehydrogenases (GLX) [42]. OAH functions in the cytosol to produce oxalic acid from oxaloacetate under appropriate conditions [42]. We found that an OAH gene Glotr_42369 was significantly upregulated on cellulose media but was downregulated on cedar media (Table 3). GLX produce oxalic acid in peroxisome. *G*. *trabeum* has two GLX genes; Glotr_54623 which was significantly upregulated on cellulose and cedar media, and Glotr_61065 which was not significantly altered in its expression level on lignocellulose media (Table 3 and S5 Table).

**Table 3:** *G*. *trabeum* oxalate and H_2_O_2_ production-associated genes.

		TPM(Average)^a^			Cel/Glc^b^		Cedar/Glc^b^			
ID	Putative function	Glc	Cel	Cedar	Ratio	Q value	Ratio	Q value	Up^c^	Down^c^
42369	Oxaloacetate acetylhydratase	573.8	3286.3	17.7	5.7	0.001	0.0	0.000	C	S
54623	Glyoxylate dehydrogenase	13.9	36.3	41.6	2.6	0.000	3.0	0.000	C, S	
139980	AA3_3 alcohol oxidase	38.9	7623.2	9168.0	195.8	0.000	235.5	0.000	C, S	
82487	AA3_2 oxidoreductase	78.7	1253.7	972.9	15.9	0.004	12.4	0.001	C, S	
89774	AA3_2 oxidoreductase	1.3	41.5	11.6	32.3	0.007	9.0	0.000	C, S	
109397	AA3_2 oxidoreductase	24.3	176.8	87.4	7.3	0.002	3.6	0.001	C, S	
130589	AA3_2 oxidoreductase	8.3	19.1	28.9	2.3	0.002	3.5	0.000	C, S	
80540	AA3_2 oxidoreductase	26.4	68.4	9.1	2.6	0.002	0.3	0.001	C	S
139690	AA3_2 oxidoreductase	18.1	133.0	58.1	7.4	0.002	3.2	0.005	C, S	
63039	AA3_2 oxidoreductase	11.7	23.9	37.1	2.0	0.012	3.2	0.000	S	
120265	AA3_2 oxidoreductase	9.9	22.1	14.9	2.2	0.000	1.5	0.001	C	
70090	AA3_2 oxidoreductase	3.9	4.7	10.4	1.2	0.169	2.7	0.000	S	
116786	AA3_4 pyranose 2-oxidase	3.4	79.3	11.7	23.1	0.006	3.4	0.001	C, S	
113732	AA3_1 cellobiose dehydrogenase	1.2	4.9	9.5	4.0	0.003	7.7	0.000	C, S	
35436	AA5_1 copper-radical oxidase	0.2	0.1	0.3	1.0	0.137	2.1	0.015		
65654	AA5_1 copper-radical oxidase	237.9	337.3	140.8	1.4	0.001	0.6	0.000		

^a^Mean TPM value for each condition (n = 3).

^b^Ratio of TPM value and Q value by LRTs between cellulose and glucose, and cedar and glucose.

^c^Genes determined as upregulated (Up) or downregulated (Down). C: Cellulose, S: Cedar.

As reviewed earlier, H_2_O_2_ production can occur by non-enzymatic redox cycling of phenolics within the wood cell wall. This chemistry is often ignored by researchers studying oxidative mechanisms in wood decay, but it is important to recognize that H_2_O_2_ production can occur via this path in addition to its production by several enzymatic sources which are known to be active during fungal decay. In this regard, the genes associated with potential enzymatic H_2_O_2_ production are quite diverse. The AA3_3 alcohol oxidase Glotr_139980, which oxidizes methanol as a preferred physiological substrate, is often discussed as a source of H_2_O_2_ in CMF reactions [16]. In our current research, Glotr_139980 was dramatically upregulated both in cellulose and cedar (20th and 9th most upregulated genes in cellulose and cedar, respectively) (Table 3). The AA3_2 family, which comprises glucose oxidase, glucose dehydrogenase, pyranose dehydrogenase, and aryl alcohol oxidase, also could be a candidate for H_2_O_2_ production [43]. In *G*. *trabeum*, five AA3_2 genes were upregulated on both cellulose and cedar media (Table 3). Two other AA3_2 genes were uniquely upregulated on cellulose media while two other AA3_2 genes were uniquely upregulated on cedar media (Table 3). AA3_4 pyranose 2-oxidase also can potentially produce extracellular H_2_O_2_ [44] and we found that the sole AA3_4 gene in *G*. *trabeum* (Glotr_116786) was upregulated on both cellulose and cedar media (Table 3). The AA3_1 enzyme family is the flavin domain of cellobiose dehydrogenase, mostly fused with the AA8 domain, which has also been reported to produce H_2_O_2_ [45]. Glotr_113732, the only AA3_1 gene in *G*. *trabeum*, lacks the AA8 domain. This gene was also upregulated on cellulose and cedar media in our work (Table 3). AA5_1 copper-radical oxidases (CROs) are H_2_O_2_-generating enzymes that have broad substrate specificity [17]. *G*. *trabeum* has two CRO2 genes (Glotr_605654 and Glotr_35436), but these genes were not determined to be DEGs when *G*. *trabeum* was grown on either cellulose or cedar media (Table 3).

#### Other remarkable DEGs

Of the 20 genes that were upregulated to the greatest degree, several came from the major facilitator superfamily (MFS) transporter genes (S7 Table). Three of these were upregulated on cellulose and five on cedar. Of these, Glotr_125226 and Glotr_77119 were upregulated on both cellulose and cedar. The precise functions of these transporters are difficult to assign based on sequence homology, although the best hit (29–37% identity at 86–95% coverage, with a bit score of 202–236) within the Swiss-Prot database is the lactose permease from *Kluyveromyces lactis* (UniProt accession number P07921).

## Discussion

In this study, we analyzed the transcriptome responses of the brown rot fungus *G*. *trabeum* when grown on cellulose or Japanese cedar (conifer) cultures to elucidate the inherent plant cell wall degrading ability of this fungus. We focused on genes that had been reported previously to be potentially involved in the CMF reaction and subsequent enzymatic saccharification reactions in wood cell wall degradation, and found unique gene induction patterns for this fungus when grown on cellulose and cedar in comparison to growth on glucose.

### Cellulose and hemicellulose degradation

Relative to enzymatic action on polysaccharides, *G*. *trabeum* was previously proposed to degrade cellulose using GH5_5 and GH12 endoglucanases because it lacked GH6 and GH7 cellobiohydrolase genes [5]. In our current research, GH5_5 and GH12 genes were dramatically upregulated when *G*. *trabeum* was grown on cedar, and especially on cellulose. GH5_5 genes were also upregulated on lignocellulose media in other brown rot fungi [46–48]. However, prior research showed that GH12 genes were not upregulated in *Rhodonia placenta* and *Wolfiporia cocos*, but *S*. *lacrymans* upregulated this family of genes in pine cultures [46–48]. Our current data, taken with that in the literature, suggests that GH12 genes are expressed under different regulations depending on brown rot species.

GH131, coding for a putative cellulose degrading β-glucanase, was upregulated on lignocellulose media by *G*. *trabeum*. Previous research also showed that *S*. *lacrymans* upregulated a GH131 gene in pine cultures (*R*. *placenta* and *W*. *cocos* do not possess this gene family) [48]. In addition, GH131 genes were also upregulated in lignocellulose media by at least two white rot fungi [49,50]. The precise function of GH131 enzymes is still unclear at present, but these results suggest the importance of this family of enzymes in lignocellulose degradation for wood decay fungi.

Among the AA9 genes, only GtLPMO9B was upregulated on lignocellulose media. GtLPMO9B was previously reported to have activity on cellulose and xyloglucan [51]. This property of GtLPMO9B is consistent with the result of upregulation on lignocellulose media. Although GtLPMO9A demonstrated significant activity against xyloglucan in prior research [30], it was downregulated on both cellulose and cedar media. GtLPMO9A is speculated to function in primary wood cell wall degradation [30] and it might function when *G*. *trabeum* attacks grasses or other plants without heavily lignified secondary cell walls. In *S*. *lacrymans*, three AA9 genes were upregulated on pine cultures [48]; however, in *R*. *placenta* and *W*. *cocos* [46,52] AA9 genes were not determined to be DEGs on lignocellulose media. To date, there have been only two brown rot AA9 enzymes that have been characterized [30,51], and these two AA9s are from *G*. *trabeum*. To better understand the role of AA9 enzymes in brown rot fungi, additional enzymatic characterization of this family of enzymes is needed.

With hemicellulases, as seen in other brown rot fungi, *G*. *trabeum* upregulated GH10 and GH5_7 genes on lignocellulose media [46,48,52]. In addition we found that the CE1 gene in *G*. *trabeum* was upregulated on lignocellulose media, which appears to be a unique new finding in *G*. *trabeum*. Presley et al. [53] suggested that the CE1 gene contributed to the grass substrate-degrading ability of *G*. *trabeum*, as *S*. *lacrymans* which does not have CE1 gene, does not efficiently degrade grasses. As we did not test on grasses, we are unable to confirm this finding; however, the upregulation of the CE1 gene on lignocellulose media suggests its importance in arabinoxylan degradation in *G*. *trabeum*.

### Biosynthesis of LMW iron-binding metabolites

As mentioned in the Results section, secondary metabolites such as quinones and catechols function as LMW chelators which have been shown to induce the CMF reaction in brown rot fungi [2,35,54]. In the present study, similarly to prior work with the brown rot *R*. *placenta*, *G*. *trabeum* showed no upregulation of PKS genes on lignocellulose media [47]. Conversely, *W*. *cocos* showed upregulation of a PKS gene on aspen and pine media [46]. Riley et al. [6] showed that brown rot fungi have a larger number of PKS genes in their genomes compared to white rot fungi. Although our data is too limited to make a definitive conclusion, our results suggest that PKS genes may not be directly related to LMW chelator action involved in wood degradation by *G*. *trabeum*. Contrary to PKS genes, TS genes were upregulated on lignocellulose media in *G*. *trabeum*. A TS gene was also upregulated on pine media in *W*. *cocos* [46], while no upregulation in TS genes was observed in *R*. *placenta* [47]. There is no direct evidence for the relatedness of TS genes in siderophore production, but the upregulated TS genes in these two brown rot fungi might be involved in the production of iron-binding chelating metabolites similar to siderophores; potentially if associated in a CYP cluster with P450 genes (below).

In this study, while many genes were upregulated both for cellulose and cedar conditions, significantly more P450 genes were upregulated on cedar than cellulose. P450 gene upregulation in coniferous cultures is a common trend in brown rot fungi [46,47]. Considering the diversity of P450 functions and the difficulty in predicting specific functions based on sequence similarity, the specific functions of these upregulated P450 genes are difficult to predict. Previous studies have suggested that P450s are involved in the degradation of lignins and terpenes [38,40,55]. The increased gene expression of the P450s on conifers in brown rot fungi suggests the involvement of the P450s in the degradation of these compounds as suggested in earlier research [46]. However, some reports which show gene clusters of P450s with TSs suggest the involvement of P450 enzymes in iron-binding metabolite biosynthesis [56]. Additional study of both the upregulated P450s and TSs found in this study may be fruitful in uncovering novel iron chelator biosynthesis pathways involving these P450s and TSs.

### Enzymes potentially involved in LMW catechol redox reactions

An AA6 quinone reductase was previously found to serve as a reductant of 2,5-DMBQ. The involvement of this metabolite in CMF reactions by *G*. *trabeum* has been suggested in the literature [12,57]. In our research, *G*. *trabeum* showed no upregulation of AA6 genes on lignocellulose media, which is not surprising given the breadth of substrates that AA6 enzymes can act on and the more common role of quinone reductases in the detoxification of quinones and reduction of oxidative stress in the extracellular environment [58,59]. We suggest that it would not be possible for an AA6 quinone reductase to diffuse deep into the wood cell wall where 2,5-DMBQ and lignin-derived compounds need to cycle to reduce iron as part of the CMF reaction. In other research, upregulation of AA6 genes also was not shown when *W*. *cocos* was grown on wood; however, an AA6 gene was upregulated on cellulose and aspen cultures in *R*. *placenta* [8,46,52]. Even though the involvement of the AA6 enzyme in *G*. *trabeum* and *W*. *cocos* CMF reactions does not appear to be strong, it is possible that it may play a role in some other brown rot degradative mechanisms.

A *G*. *trabeum* AA1_1 laccase gene was upregulated on lignocellulose media similarly to that previously reported in *R*. *placenta* and *W*. *cocos* [46,52]. As with the AA6 enzymes (and all known extracellular enzymes), AA1_1 enzymes would be too large to penetrate deep into the wood cell wall. This may suggest AA1_1 works in the extracellular matrix (ECM) surrounding the fungal hyphae to initially oxidize fungal hydroquinone metabolites to semiquinones to permit the semiquinones, with their better iron-reduction potential, to then diffuse into the wood cell wall. This role in the fungal ECM would also extend to detoxification of other phenolic compounds to protect the fungus.

*G*. *trabeum* also upregulated putative Fe^3+^-reducing GLP genes on cellulose and cedar media similar to that found with *R*. *placenta* and *W*. *cocos* when grown on wood substrates[46,52]. The estimated size of these gene products was ca. 13–24 kDa, and would be much larger than could penetrate the intact wood cell wall. We therefore propose that it is unlikely that these products are involved directly in brown rot action on the wood cell wall as has also been previously discussed [60]. The physiological role of GLP is obscure, but the upregulation in lignocellulose cultures in brown rot fungi suggests a function related in some other manner to wood degradation processes.

### Oxalate and H_2_O_2_ production

Relative to oxalic acid production by *G*. *trabeum*, an OAH gene was upregulated on cellulose media but downregulated on cedar media. In prior research with *R*. *placenta* and *W*. *cocos*, OAH genes were not upregulated on lignocellulose media [46,47]. In our work, however, a GLX gene was upregulated on lignocellulose media in *G*. *trabeum*. GLX genes were also upregulated on aspen and pine in *W*. *cocos*, but not upregulated in *R*. *placenta* [46,47]. This suggests that oxalic acid production by GLX is induced on lignocellulose media, at least in these two fungi. Zhuang et al. [42] showed that in ^13^C metabolic flux experiments, *G*. *trabeum* predominantly used the OAH pathway for oxalic acid production under low C/N conditions, while the GLX pathway predominated under high C/N conditions. However, qPCR results showed no significant difference in the gene expression levels when comparing the two C/N conditions. Wood is a low nitrogen environment and our data support that growth of *G*. *trabeum* on high C/N substrates like wood induces GLX gene expression.

We also examined several oxidase genes related to H_2_O_2_ production in this study. *G*. *trabeum* dramatically upregulated AA3_3 methanol oxidase and also upregulated AA3_1, AA3_2, and AA3_4 genes when on cellulose and cedar; however, AA5_1 genes were not upregulated. AA3_3 genes were also previously shown to be highly upregulated in other brown and white rot fungi when on wood substrates [46,52,61,62] suggesting the importance of this family of enzymes in wood degradation processes. AA3_1 and AA3_4 genes are not generally present in brown rot fungi [6] and AA3_2 genes have been shown not to be upregulated in other brown rot fungi [46,52]. However AA5_1 genes were shown to be upregulated in other brown rot fungi such as *R*. *placenta* and *W*. *cocos* [46,52]. This suggests that these enzymes may play a role in differential substrate degradation properties of these brown rot fungi. Considering that these enzymes possibly produce H_2_O_2_ in the fungal ECM, the role of enzymatically-generated H_2_O_2_ in *G*. *trabeum* decay is uncertain. However, it is clear that with the large number of genes that are upregulated for H_2_O_2_ biosynthesis, the role or roles played by H_2_O_2_ appear to be important to the fungus, particularly during wood decay.

### Limitations of this research and current transcriptomic analyses

Zhang et al. [4] demonstrated the importance of temporal analysis for the study of brown rot mechanisms by RNA-seq analysis of three sequential decay stages of mycelia. Wu et al. [63] further discussed the importance of gene expression and gene editing corresponding to the degradation of different woody substrates in both brown rot and white rot fungi. The gene expression profiles of the brown rot fungus discussed in the current study are based on a single time point data and with a limited substrate selection. We used different cultivation periods for glucose and lignocellulose substrates (glucose-3 days, cellulose and cedar-5 days) to adjust for the different growth stages of the fungus on these substrates. On glucose *G*. *trabeum* grows quickly, but it undergoes autolysis after 5 days. On wood substrates however, *G*. *trabeum* grows adqeuately for analysis only after 5 days. Therefore, the differences in gene expression patterns noted provide useful data, but we recognize that they allow only a limited comparison of the *G*. *trabeum* transcriptome to that of other degradative fungi on a diverse array of substrates. To detail the differences between brown rot fungal wood decay species, future research must focus on the analysis of multiple time point transcriptomes using additional substrates with different degrees of lignification, and also with wood substrates containing different levels and types of wood extractives and resins. Furthermore, it is important to recognize that all enzymes expressed by fungi may not have specific roles, and at least some gene products from fungi must be considered vestigial, pseudo-enzymes in nature, similar to those found in other organisms [64]. We recognize the importance of exploration of the genome for new enzymes and enzyme expression patterns in fungi, but also recognize that without linkage of secretion to actual function via degradation analyses, that much additional research remains to be conducted. The research we present in this paper provides initial findings along that path for brown rot fungi.

## Conclusion

In this study, we performed RNA-seq analysis of the well-studied brown rot fungus *G*. *trabeum* cultured on lignocellulose media. In contrast to other brown rot fungi such as *R*. *placenta* and *W*. *cocos*, *G*. *trabeum* upregulated GH12, AA9 and AA3_2 genes in lignocellulose media. In addition, unlike other brown rot fungi, *G*. *trabeum* possesses GH131, CE1, AA3_1, and AA3_4 genes, and these genes also were upregulated on lignocellulose media. These results suggest the contribution of these genes in the inherent plant substrate assimilation properties of *G*. *trabeum*. Conversely, however, AA6 and AA5_1 genes, which were upregulated in *R*. *placenta*, were not upregulated in *G*. *trabeum*. The upregulation of both TS and P450 genes suggests the potential importance of these genes in the production of secondary metabolites that should be explored further for the potential of gene products in brown rot CMF reactions.

These different gene expression patterns provide clues that may help to clarify the basis for different lignocellulose-degrading capacities and substrate selection by different brown rot fungal species. This study provides new insights into the inherent lignocellulose-degrading ability of *G*. *trabeum* and the diversity of the brown rot mechanisms, but further characterization of lignocellulose-upregulated genes will help to clarify novel factors leading to the elucidation of brown rot mechanisms.

## Supporting information

S1 Table Summary of sample information and transcriptome sequencing output statistics for the RNA-seq libraries. (DOCX)

S2 Table Transcriptome expression levels of all *G*. *trabeum* genes. (XLSX)

S3 Table *G*. *trabeum* genes encoding putative cellulose and hemicellulose degrading enzymes that were not upregulated on lignocellulose media. (DOCX)

S4 Table *G*. *trabeum* genes potentially involved in LMW iron-binding metabolite synthesis that were not upregulated on lignocellulose media. (DOCX)

S5 Table *G*. *trabeum* genes previously reported as potentially involved in LMW catechol redox reactions and the production of oxalate and H_2_O_2_, but that were not upregulated on lignocellulose media. (DOCX)

S6 Table *G*. *trabeum* genes encoding cytochrome P450 that were upregulated on cellulose and/or cedar. (DOCX)

S7 Table 20 *G*. *trabeum* genes with the greatest upregulation on cellulose or cedar. (A) Cellulose, (B) Cedar.
(DOCX)
