# Supplementary material for: Transcriptome analysis of the brown rot fungus Gloeophyllum trabeum during lignocellulose degradation
Source: PLoS One. 2020 Dec 14;15(12):e0243984. doi: 10.1371/journal.pone.0243984 (PMC7735643; doi:10.1371/journal.pone.0243984)
Supplement: S1 Table — (DOCX) [file pone.0243984.s001.docx]

**S1 Table.** Summary of sample information and transcriptome sequencing output statistics for the RNA-seq libraries.

| Growth condition | Replicate | Raw reads | Reads after quality control | Reads pseudoaligned |
| --- | --- | --- | --- | --- |
| Glucose | 1 | 14,254,120 | 13,667,141 (95.9%) | 10,054,610 (73.6%) |
|  | 2 | 14,711,744 | 14,121,090 (96.0%) | 10,305,832 (73.0%) |
|  | 3 | 17,958,770 | 17,350,782 (96.6%) | 13,556,464 (78.1%) |
| Cellulose | 1 | 24,832,769 | 24,201,887 (97.5%) | 14,832,201 (61.3%) |
|  | 2 | 19,980,805 | 19,489,030 (97.5%) | 12,812,884 (65.7%) |
|  | 3 | 18,186,261 | 17,759,800 (97.7%) | 11,310,370 (63.7%) |
| Cedar | 1 | 20,905,997 | 20,290,420 (97.1%) | 14,029,414 (69.1%) |
|  | 2 | 21,315,558 | 20,697,318 (97.1%) | 15,079,412 (72.9%) |
|  | 3 | 23,935,005 | 23,264,486 (97.2%) | 15,893,094 (68.3%) |
