# Supplementary material for: Transcriptome analysis of the brown rot fungus Gloeophyllum trabeum during lignocellulose degradation
Source: PLoS One. 2020 Dec 14;15(12):e0243984. doi: 10.1371/journal.pone.0243984 (PMC7735643; doi:10.1371/journal.pone.0243984)
Supplement: S3 Table — (DOCX) [file pone.0243984.s003.docx]

**S3 Table.** *G. trabeum* genes encoding putative cellulose and hemicellulose degrading enzymes that were not upregulated on lignocellulose media.

|  |  | TPM(Average)^a^ | | | Cel/Glc^b^ | | Cedar/Glc^b^ | |  |  |
| --- | --- | --- | --- | --- | --- | --- | --- | --- | --- | --- |
| ID | Putative function | Glc | Cel | Cedar | Ratio | Q value | Ratio | Q value | Up^c^ | Down^c^ |
| 69702 | AA9 lytic polysaccharide monooxygenase | 30.6 | 44.7 | 52.2 | 1.5 | 0.034 | 1.7 | 0.021 |  |  |
| 103762 | AA9 lytic polysaccharide monooxygenase | 1.8 | 0.7 | 2.2 | 0.4 | 0.027 | 1.3 | 0.055 |  |  |
| 12971 | GH3 exo-β-glycosidase | 29.1 | 21.6 | 16.2 | 0.7 | 0.034 | 0.6 | 0.026 |  |  |
| 75899 | GH3 exo-β-glycosidase | 105.1 | 158.9 | 38.9 | 1.5 | 0.003 | 0.4 | 0.000 |  | S |
| 138774 | GH3 exo-β-glycosidase | 72.7 | 49.0 | 34.2 | 0.7 | 0.042 | 0.5 | 0.012 |  |  |
| 49126 | GH1 β-glucosidase | 4.3 | 1.9 | 4.4 | 0.4 | 0.026 | 1.0 | 0.185 |  |  |
| 49308 | GH1 β-glucosidase | 3.9 | 1.7 | 4.9 | 0.4 | 0.018 | 1.3 | 0.013 |  |  |
| 81012 | GH1 β-glucosidase | 233.5 | 141.3 | 13.1 | 0.6 | 0.170 | 0.1 | 0.000 |  | S |
| 106990 | GH1 β-glucosidase | 0.4 | 0.4 | 0.9 | 1.0 | 0.439 | 2.2 | 0.126 |  |  |
| 138785 | GH10 β-1,4-xylanase | 1.6 | 1.6 | 2.4 | 1.1 | 0.503 | 1.5 | 0.013 |  |  |
| 50873 | GH28 endopolygalacturonase | 1.3 | 1.8 | 2.4 | 1.4 | 0.061 | 1.8 | 0.020 |  |  |
| 110574 | GH28 endopolygalacturonase | 1.4 | 0.7 | 2.3 | 0.5 | 0.026 | 1.6 | 0.150 |  |  |
| 117232 | GH28 endopolygalacturonase | 45.6 | 14.8 | 22.4 | 0.3 | 0.016 | 0.5 | 0.001 |  | S |
| 120615 | GH28 endopolygalacturonase | 0.2 | 0.1 | 0.3 | 0.8 | 0.103 | 1.6 | 0.020 |  |  |
| 122631 | GH28 endopolygalacturonase | 0.7 | 0.5 | 1.1 | 0.7 | 0.072 | 1.5 | 0.013 |  |  |
| 140071 | GH28 endopolygalacturonase | 1.4 | 1.0 | 0.8 | 0.7 | 0.078 | 0.6 | 0.020 |  |  |
| 112531 | CE8 pectin metylesterase | 1.0 | 0.8 | 2.7 | 0.8 | 0.577 | 2.6 | 0.018 |  |  |

# ^a^Mean TPM value for each condition (n=3).

^b^Ratio of TPM value and Q value by LRTs between cellulose and glucose, and cedar and glucose.

^c^Genes determined as upregulated (Up) or downregulated (Down). C: cellulose, S: cedar.
