# Supplementary material for: Transcriptome analysis of the brown rot fungus Gloeophyllum trabeum during lignocellulose degradation
Source: PLoS One. 2020 Dec 14;15(12):e0243984. doi: 10.1371/journal.pone.0243984 (PMC7735643; doi:10.1371/journal.pone.0243984)
Supplement: S4 Table — (DOCX) [file pone.0243984.s004.docx]

**S4 Table.** *G. trabeum* genes potentially involved in LMW iron-binding metabolite synthesis that were not upregulated on lignocellulose media.

|  |  | TPM(Average)^a^ | | | Cel/Glc^b^ | | Cedar/Glc^b^ | |  |  |
| --- | --- | --- | --- | --- | --- | --- | --- | --- | --- | --- |
| ID | Putative function | Glc | Cel | Cedar | Ratio | Q value | Ratio | Q value | Up^c^ | Down^c^ |
| 91951 | Polyketide synthase (PKS4^d^) | 22.1 | 7.5 | 6.8 | 0.3 | 0.004 | 0.3 | 0.001 |  | C, S |
| 103847 | Polyketide synthase (PKS2^d^) | 2.5 | 3.2 | 4.8 | 1.3 | 0.088 | 1.9 | 0.000 |  |  |
| 116317 | Polyketide synthase (PKS1^d^) | 104.3 | 16.7 | 3.2 | 0.2 | 0.000 | 0.0 | 0.000 |  | C, S |
| 140669 | Polyketide synthase (PKS7^d^) | 4.9 | 3.2 | 5.5 | 0.7 | 0.085 | 1.1 | 0.015 |  |  |
| 47645 | Terpene synthase | 5.5 | 1.1 | 3.5 | 0.2 | 0.002 | 0.6 | 0.024 |  | C |
| 48290 | Terpene synthase | 4.0 | 1.6 | 2.2 | 0.4 | 0.004 | 0.6 | 0.008 |  | C |
| 64172 | Terpene synthase | 121.4 | 189.9 | 31.8 | 1.6 | 1.000 | 0.3 | 0.000 |  | S |
| 78472 | Terpene synthase | 2.5 | 3.0 | 2.5 | 1.2 | 0.146 | 1.0 | 0.029 |  |  |
| 117180 | Terpene synthase | 75.8 | 117.1 | 72.3 | 1.5 | 0.954 | 0.3 | 1.000 |  |  |
| 117331 | Terpene synthase | 162.4 | 261.5 | 49.8 | 1.6 | 1.000 | 0.3 | 0.000 |  | S |

# ^a^Mean TPM value for each condition (n=3).

^b^Ratio of TPM value and Q value by LRTs between cellulose and glucose, and cedar and glucose.

^c^Genes determined as upregulated (Up) or downregulated (Down). C: cellulose, S: cedar.

^d^Lackner G, Misiek M, Braesel J, Hoffmeister D. Genome mining reveals the evolutionary origin and biosynthetic potential of basidiomycete polyketide synthases. Fungal Genet. Biol. 2012; 49(12)**,** 996–1003. http://dx.doi.org/﻿10.1016/j.fgb.2012.09.009. PMID: 23078836
