# Supplementary material for: Transcriptome analysis of the brown rot fungus Gloeophyllum trabeum during lignocellulose degradation
Source: PLoS One. 2020 Dec 14;15(12):e0243984. doi: 10.1371/journal.pone.0243984 (PMC7735643; doi:10.1371/journal.pone.0243984)
Supplement: S5 Table — (DOCX) [file pone.0243984.s005.docx]

**S5 Table.** *G. trabeum* genes previously reported as potentially involved in LMW catechol redox reactions and the production of oxalate and H_2_O_2_, but that were not upregulated on lignocellulose media.

|  |  | TPM(Average)^a^ | | | Cel/Glc^b^ | | Cedar/Glc^b^ | |  |  |
| --- | --- | --- | --- | --- | --- | --- | --- | --- | --- | --- |
| ID | Putative function | Glc | Cel | Cedar | Ratio | Q value | Ratio | Q value | Up^c^ | Down^c^ |
| 43770 | AA1_1 laccase | 2.9 | 1.7 | 4.5 | 0.6 | 0.013 | 1.6 | 0.005 |  |  |
| 107459 | AA1_1 laccase | 1.8 | 0.7 | 2.3 | 0.4 | 0.003 | 1.3 | 0.013 |  | C |
| 127593 | AA1_1 laccase | 4.3 | 3.3 | 8.5 | 0.8 | 0.142 | 2.0 | 0.005 |  |  |
| 61065 | Glyoxylate dehydrogenase | 19.0 | 34.5 | 16.5 | 1.8 | 0.001 | 0.9 | 0.019 |  |  |
| 106260 | AA3_3 alcohol oxidase | 6.8 | 8.1 | 3.3 | 1.2 | 1.000 | 0.5 | 0.148 |  |  |

# ^a^Mean TPM value for each condition (n=3).

^b^Ratio of TPM value and Q value by LRTs between cellulose and glucose, and cedar and glucose.

^c^Genes determined as upregulated (Up) or downregulated (Down). C: cellulose, S: cedar.
