# Supplementary material for: Transcriptome analysis of the brown rot fungus Gloeophyllum trabeum during lignocellulose degradation
Source: PLoS One. 2020 Dec 14;15(12):e0243984. doi: 10.1371/journal.pone.0243984 (PMC7735643; doi:10.1371/journal.pone.0243984)
Supplement: S6 Table — (DOCX) [file pone.0243984.s006.docx]

**S6 Table.** *G. trabeum* genes encoding cytochrome P450 that were upregulated on cellulose and/or cedar.

|  |  | TPM(Average)^a^ | | | Cel/Glc^b^ | | Cedar/Glc^b^ | |  |  |
| --- | --- | --- | --- | --- | --- | --- | --- | --- | --- | --- |
| ID | KOG define | Glc | Cel | Cedar | Ratio | Q value | Ratio | Q value | Up^c^ | Down^c^ |
| 31501 | CYP2 | 6.0 | 8.7 | 14.6 | 1.5 | 0.008 | 2.4 | 0.000 | S |  |
| 42383 | CYP2 | 1.2 | 1.6 | 2.6 | 1.3 | 0.059 | 2.1 | 0.001 | S |  |
| 44705 | CYP2 | 2.5 | 2.1 | 8.0 | 0.9 | 0.097 | 3.2 | 0.000 | S |  |
| 45842 | CYP2 | 3.6 | 2.1 | 9.1 | 0.6 | 0.036 | 2.6 | 0.000 | S |  |
| 45946 | CYP3/CYP5/CYP6/CYP9 | 1.2 | 1.1 | 3.8 | 0.9 | 0.143 | 3.2 | 0.000 | S |  |
| 46298 | CYP2 | 23.0 | 32.7 | 67.5 | 1.4 | 0.004 | 2.9 | 0.000 | S |  |
| 48505 | CYP2 | 23.5 | 451.9 | 335.6 | 19.2 | 0.015 | 14.3 | 0.004 | S |  |
| 48978 | CYP2 | 0.8 | 2.0 | 5.7 | 2.6 | 0.027 | 7.4 | 0.000 | S |  |
| 50230 | CYP4/CYP19/CYP26 | 21.1 | 51.4 | 57.1 | 2.4 | 0.014 | 2.7 | 0.000 | S |  |
| 50598 | CYP2 | 6.2 | 4.8 | 16.7 | 0.8 | 0.402 | 2.7 | 0.000 | S |  |
| 82138 | NA (CYP4/CYP19/CYP26 ) ^d^ | 0.1 | 0.4 | 0.5 | 5.2 | NA | 6.2 | 0.003 | S |  |
| 82372 | CYP4/CYP19/CYP26 | 5.3 | 8.5 | 18.3 | 1.6 | 0.116 | 3.4 | 0.000 | S |  |
| 94923 | CYP2 | 0.6 | 0.5 | 2.4 | 0.8 | 0.520 | 3.9 | 0.004 | S |  |
| 107059 | CYP2 | 3.0 | 3.7 | 8.4 | 1.3 | 0.428 | 2.8 | 0.003 | S |  |
| 116311 | CYP4/CYP19/CYP26 | 1.0 | 0.6 | 4.2 | 0.6 | 0.052 | 4.3 | 0.000 | S |  |
| 120247 | CYP2 | 4.6 | 3.7 | 15.9 | 0.8 | 0.024 | 3.4 | 0.000 | S |  |
| 123943 | CYP2 | 2.0 | 4.5 | 4.1 | 2.2 | 0.166 | 2.0 | 0.001 | S |  |
| 127266 | CYP2 | 5.3 | 2.8 | 11.0 | 0.5 | 0.008 | 2.1 | 0.001 | S |  |
| 128802 | CYP2 | 1.8 | 1.5 | 5.9 | 0.8 | 0.087 | 3.2 | 0.000 | S |  |
| 133732 | CYP4/CYP19/CYP26 | 3.9 | 2.3 | 9.9 | 0.6 | 0.038 | 2.5 | 0.000 | S |  |
| 140036 | CYP3/CYP5/CYP6/CYP9 | 9.6 | 4.8 | 65.8 | 0.5 | 0.002 | 6.9 | 0.000 | S |  |
| 9976 | CYP2 | 3.2 | 18.1 | 27.7 | 5.7 | 0.002 | 8.7 | 0.000 | C, S |  |
| 30236 | CYP4/CYP19/CYP26 | 0.6 | 3.0 | 2.1 | 4.8 | 0.005 | 3.3 | 0.001 | C, S |  |
| 44427 | CYP4/CYP19/CYP26 | 0.7 | 10.4 | 13.4 | 14.0 | 0.000 | 17.9 | 0.000 | C, S |  |
| 63933 | CYP2 | 0.9 | 14.3 | 62.8 | 16.0 | 0.000 | 70.2 | 0.000 | C, S |  |
| 80617 | CYP3/CYP5/CYP6/CYP9 | 7.7 | 33.9 | 61.3 | 4.4 | 0.000 | 8.0 | 0.000 | C, S |  |
| 124807 | CYP4/CYP19/CYP26 | 2.3 | 8.3 | 30.5 | 3.6 | 0.000 | 13.2 | 0.000 | C, S |  |
| 132312 | CYP3/CYP5/CYP6/CYP9 | 5.3 | 103.6 | 75.8 | 19.4 | 0.000 | 14.2 | 0.000 | C, S |  |
| 138963 | CYP4/CYP19/CYP26 | 5.1 | 47.1 | 50.0 | 9.2 | 0.000 | 9.7 | 0.000 | C, S |  |
| 140224 | CYP2 | 11.9 | 932.8 | 529.4 | 78.3 | 0.000 | 44.4 | 0.000 | C, S |  |
| 140284 | CYP2 | 1.8 | 12.3 | 15.2 | 6.6 | 0.000 | 8.2 | 0.000 | C, S |  |
| 43580 | CYP2 | 6.3 | 20.0 | 15.6 | 3.2 | 0.005 | 2.5 | 0.017 | C |  |
| 112374 | CYP2 | 95.6 | 301.6 | 146.7 | 3.2 | 0.000 | 1.5 | 0.016 | C |  |
| 115907 | CYP4/CYP19/CYP26 | 15.2 | 38.5 | 24.8 | 2.5 | 0.000 | 1.6 | 0.006 | C |  |
| 132663 | CYP4/CYP19/CYP26 | 20.5 | 42.2 | 29.5 | 2.1 | 0.004 | 1.4 | 0.006 | C |  |

# ^a^Mean TPM value for each condition (n=3).

^b^Ratio of the TPM value and Q value by LRTs between cellulose and glucose, and cedar and glucose.

^c^Genes determined as upregulated (Up) or downregulated (Down). C: cellulose, S: cedar.

^d^NA: not applicable. The annotations in parenthesis are the results from a Conserved Domain Search service (https://www.ncbi.nlm.nih.gov/Structure/cdd/wrpsb.cgi) (﻿Marchler-Bauer A, Bo Y, Han L, He J, Lanczycki CJ, Lu S, et al. CDD/SPARCLE: Functional classification of proteins via domain architectures. Nucleic Acids Res. 2017; 45:D200–D203. doi:10.1093/nar/gkw1129. PMID: 27899674).
