# Supplementary material for: Transcriptome analysis of the brown rot fungus Gloeophyllum trabeum during lignocellulose degradation
Source: PLoS One. 2020 Dec 14;15(12):e0243984. doi: 10.1371/journal.pone.0243984 (PMC7735643; doi:10.1371/journal.pone.0243984)
Supplement: S7 Table — (A) Cellulose, (B) Cedar. (DOCX) [file pone.0243984.s007.docx]

**S7A Table.** 20 *G. trabeum* genes with the greatest upregulation on cellulose.

|  |  | TPM(Average)^a^ | | | Cel/Glc^b^ | | Cedar/Glc^b^ | |  |  |
| --- | --- | --- | --- | --- | --- | --- | --- | --- | --- | --- |
| ID | Define | Glc | Cel | Cedar | Ratio | Q value | Ratio | Q value | Up^c^ | Down^c^ |
| 46670 | Alcohol dehydrogenase, class V | 0.0 | 147.3 | 0.0 | 1.7×10^8^ | 0.001 | 377.7 | - | C |  |
| 130898 | hypothetical protein | 15.2 | 49956.2 | 14113.2 | 3296.2 | 0.000 | 931.2 | 0.000 | C, S |  |
| 63180 | GH5_5 endo-ß-1,4-glucanase | 3.4 | 8279.2 | 246.0 | 2401.3 | 0.000 | 71.4 | 0.002 | C, S |  |
| 126265 | hypothetical protein | 1.7 | 2511.2 | 2568.1 | 1437.4 | 0.000 | 1470.0 | 0.000 | C, S |  |
| 125226 | MFS transporter | 0.2 | 159.2 | 327.4 | 886.4 | 0.000 | 1822.9 | 0.000 | C, S |  |
| 138821 | GH12 endo-ß-1,4-glucanase | 4.8 | 3698.0 | 62.3 | 772.8 | 0.000 | 13.0 | 0.001 | C, S |  |
| 77119 | MFS transporter | 3.0 | 2078.5 | 519.9 | 697.8 | 0.000 | 174.5 | 0.000 | C, S |  |
| 45607 | GH5_22 ß-glycanase | 0.0 | 30.8 | 1.0 | 641.1 | 0.000 | 20.7 | 0.006 | C, S |  |
| 77637 | CE10 esterase | 0.6 | 349.0 | 165.6 | 548.1 | 0.000 | 260.1 | 0.000 | C, S |  |
| 140289 | CBM1-GH10  β-1,4-xylanase | 2.2 | 1026.3 | 138.0 | 456.5 | 0.000 | 61.4 | 0.002 | C, S |  |
| 80788 | GH109 N-acetylgalactosaminidase | 0.0 | 15.7 | 6.7 | 380.4 | 0.001 | 161.7 | 0.000 | C, S |  |
| 110321 | hypothetical protein | 7.6 | 2578.1 | 634.4 | 338.1 | 0.000 | 83.2 | 0.000 | C, S |  |
| 46499 | GH10 β-1,4-xylanase | 6.9 | 2320.5 | 106.5 | 337.1 | 0.000 | 15.5 | 0.003 | C, S |  |
| 131089 | Short-chain alcohol dehydrogenase | 0.5 | 146.2 | 102.4 | 323.8 | 0.000 | 226.7 | 0.000 | C, S |  |
| 63531 | AA9 lytic polysaccharide monooxygenase | 27.2 | 8353.6 | 798.7 | 306.6 | 0.000 | 29.3 | 0.005 | C, S |  |
| 117176 | MFS transporter | 0.5 | 134.8 | 21.7 | 252.5 | 0.000 | 40.7 | 0.000 | C, S |  |
| 137245 | hypothetical protein | 7.6 | 1845.8 | 1327.8 | 241.3 | 0.000 | 173.6 | 0.000 | C, S |  |
| 96525 | hypothetical protein | 0.9 | 218.3 | 79.1 | 235.6 | 0.000 | 85.3 | 0.000 | C, S |  |
| 141454 | hypothetical protein | 1.4 | 279.9 | 127.3 | 198.2 | 0.000 | 90.1 | 0.000 | C, S |  |
| 139980 | AA3_3 alcohol oxidase | 38.9 | 7623.2 | 9168.0 | 195.8 | 0.000 | 235.5 | 0.000 | C, S |  |

# ^a^Mean TPM value for each condition (n=3).

^b^Ratio of the TPM value and Q value by LRTs between cellulose and glucose, and cedar and glucose.

^c^Genes determined as upregulated (Up) or downregulated (Down). C: cellulose, S: cedar.

**S7B Table.** 20 *G. trabeum* genes with the greatest upregulation on cedar.

|  |  | TPM(Average)^a^ | | | Cel/Glc^b^ | | Cedar/Glc^b^ | |  |  |
| --- | --- | --- | --- | --- | --- | --- | --- | --- | --- | --- |
| ID | Define | Glc | Cel | Cedar | Ratio | Q value | Ratio | Q value | Up^c^ | Down^c^ |
| 125226 | MFS transporter | 0.2 | 159.2 | 327.4 | 886.4 | 0.000 | 1822.9 | 0.000 | C, S |  |
| 126265 | hypothetical protein | 1.7 | 2511.2 | 2568.1 | 1437.4 | 0.000 | 1470.0 | 0.000 | C, S |  |
| 130898 | hypothetical protein | 15.2 | 49956.2 | 14113.2 | 3296.2 | 0.000 | 931.2 | 0.000 | C, S |  |
| 130130 | hypothetical protein | 0.4 | 74.3 | 342.9 | 194.5 | 0.000 | 898.0 | 0.000 | C, S |  |
| 82071 | AA4 vanillyl-alcohol oxidase | 0.1 | 1.9 | 15.4 | 36.8 | 0.000 | 295.6 | 0.000 | C, S |  |
| 77637 | CE10 esterase | 0.6 | 349.0 | 165.6 | 548.1 | 0.000 | 260.1 | 0.000 | C, S |  |
| 104526 | hypothetical protein | 1.1 | 197.4 | 277.5 | 181.3 | 0.001 | 254.8 | 0.000 | C, S |  |
| 139707 | MFS transporter | 1.8 | 212.2 | 434.0 | 115.5 | 0.000 | 236.3 | 0.000 | C, S |  |
| 139980 | AA3_3 alcohol oxidase | 38.9 | 7623.2 | 9168.0 | 195.8 | 0.000 | 235.5 | 0.000 | C, S |  |
| 131089 | Short-chain alcohol dehydrogenase | 0.5 | 146.2 | 102.4 | 323.8 | 0.000 | 226.7 | 0.000 | C, S |  |
| 73346 | 15-hydroxyprostaglandin dehydrogenase | 0.5 | 23.1 | 102.7 | 49.3 | 0.000 | 219.3 | 0.000 | C, S |  |
| 77119 | MFS transporter | 3.0 | 2078.5 | 519.9 | 697.8 | 0.000 | 174.5 | 0.000 | C, S |  |
| 133009 | hypothetical protein | 1.3 | 54.0 | 224.4 | 42.0 | 0.000 | 174.2 | 0.000 | C, S |  |
| 137245 | hypothetical protein | 7.6 | 1845.8 | 1327.8 | 241.3 | 0.000 | 173.6 | 0.000 | C, S |  |
| 40998 | Short-chain alcohol dehydrogenase | 3.3 | 538.4 | 547.3 | 164.6 | 0.000 | 167.3 | 0.000 | C, S |  |
| 122499 | MFS transporter | 2.3 | 15.1 | 373.7 | 6.6 | 0.000 | 162.6 | 0.000 | C, S |  |
| 80788 | GH109 N-acetylgalactosaminidase | 0.0 | 15.7 | 6.7 | 380.4 | 0.001 | 161.7 | 0.000 | C, S |  |
| 141070 | hypothetical protein | 2.2 | 126.0 | 279.1 | 57.0 | 0.000 | 126.2 | 0.000 | C, S |  |
| 137467 | short-chain alcohol dehydrogenase | 3.8 | 54.6 | 436.9 | 14.3 | 0.017 | 114.0 | 0.000 | S |  |
| 131603 | MFS transporter | 0.8 | 35.3 | 84.8 | 46.5 | 0.000 | 111.7 | 0.000 | C, S |  |

# ^a^Mean TPM value for each condition (n=3).

^b^Ratio of the TPM value and Q value by LRTs between cellulose and glucose, and cedar and glucose.

^c^Genes determined as upregulated (Up) or downregulated (Down). C: cellulose, S: cedar.
